# Typhoid fever and COVID-19 pandemic in Nigeria: a call for coordinated action

**DOI:** 10.31744/einstein_journal/2021CE6796

**Published:** 2021-12-15

**Authors:** Esther Edet Bassey, Mohammad Mehedi Hasan, Ana Carla dos Santos Costa, Christos Tsagkaris, Abdullahi Tunde Aborode, Ayah Karra-Aly, Mohammad Yasir Essar, Shoaib Ahmad

**Affiliations:** 1 Faculty of Basic Medical Sciences University of Uyo Uyo NG Nigeria Faculty of Basic Medical Sciences, University of Uyo, Uyo, NG, Nigeria.; 2 Faculty of Life Science Mawlana Bhashani Science and Technology University Tangail BD Bangladesh Faculty of Life Science, Mawlana Bhashani Science and Technology University, Tangail, BD, Bangladesh.; 3 Faculdade de Medicina Universidade Federal da Bahia Salvador BA Brazil Faculdade de Medicina, Universidade Federal da Bahia, Salvador, BA, Brazil.; 4 School of Medicine University of Crete Heraklion GR Greece School of Medicine, University of Crete, Heraklion, GR, Greece.; 5 Healthy Africans Platform, Research and Development Ibadan NG Nigeria Healthy Africans Platform, Research and Development, Ibadan, NG, Nigeria.; 6 Schulich School of Medicine and Dentistry University of Western Ontario London CA Canada Schulich School of Medicine and Dentistry, University of Western Ontario, London, CA, Canada.; 7 Medical Research Center Kateb University Kabul AF Afghanistan Medical Research Center, Kateb University, Kabul, AF, Afghanistan.; 8 Punjab Medical College Faisalabad PK Pakistan Punjab Medical College, Faisalabad, PK, Pakistan.

Dear Editor,

Typhoid fever, also known as enteric fever, is a significant public health issue in low- and middle-income countries (LMICs).^( 1 )^ According to the World Health Organization (WHO), approximately 11 to 20 million people across the world fall ill because of typhoid, with mortality of 128,000 to 161,000 individuals each year.^( 2 )^ The disease, caused by the *Gram* -negative bacteria *Salmonella typhi* , is transmitted through the oral consumption of a pathogenic portion of the bacterium, most often via infected food or water.^( 3 )^ It can also be spread through personal contact due to unsanitary practices and contamination of water supply.^( 4 )^

Due to the ways in which the disease is transmitted, LMICs with water sanitation problems, poor living conditions, and unhygienic practices, are more susceptible to typhoid fever disease outbreaks.^( 4 )^ In Nigeria, we can underline some factors, such as regional migration of workers, lack of proper sewage management system, inadequate supply of drinkable water, high rate of rural to urban area migration, and insufficient health facilities.^( 5 )^

Due to the absence of a country-wide epidemiological surveillance system, inadequate data availability, and restricted laboratory capacity, it is difficult to assess the prevalence of typhoid fever in Nigeria. This underreporting is also caused by factors, such as the administration of antibiotics before confirmatory laboratory test, and the fact patients do not always seek health care treatment at hospitals, or use the laboratory services, since they are not free. However, past study has documented a prevalence between 0.071%, in Oyo, and 47.1%, in Osun.^( 5 )^

In addition to providing access to potable water and improving sanitation and health facilities, vaccination against the disease is one of the best ways to combat typhoid fever. Currently there are two international commercially available vaccines for the disease, which is safe for use in children, the group at higher risk of infection by *S. typhi* . In fact, it has been shown the use of typhoid vaccines in children older than 2 years can decrease the risk of transmission of the disease.^( 6 )^ Despite the advantages, the typhoid vaccines have not been incorporated into the Nigerian Expanded Program on Immunization, accounting for an extra burden in the fight against the disease.^( 5 )^

As expected, the coronavirus disease 2019 (COVID-19) pandemic had a negative impact on the control of infectious diseases around the world, especially in exhausting health-care delivery systems.^( 7 - 9 )^ This happened mainly due to the increased demand for human resources, laboratories and infrastructure during the COVID-19 pandemic, which diverted efforts previously aimed to other diseases. In Nigeria, the situation was no different. Until April 30, 2021, 164,993 confirmed cases, with 2,063 deaths were recorded in the country.^( 10 )^ In an attempt to combat the disease, the Nigerian government adopted worldwide approved strategies, such as lockdowns, use of facemasks, social distancing and hand-washing. However, as effective as these approaches are, there has been a negligence in the fight against numerous of pre-existing infectious diseases in the country, and the pandemic increased the already existing burden on the country’s health system.^( 11 )^

The situation is more challenging, since several symptoms of typhoid fever being similar to the clinical presentation COVID-19.^( 8 , 12 )^ For instance, typhoid fever usually presents with symptoms ranging from slow onset of continuous fever, chills, liver and spleen enlargement (hepatosplenomegaly) and abdominal pain, to anorexia, rash, headache, diarrhea or constipation, nausea, relative bradycardia and low consciousness level.^( 12 )^ In comparison, COVID-19 symptoms include fever, headache, shortness of breath, arthralgia, sore throat, fatigue, chest pain, hypogeusia, hyposmia, cough, amongst others.^( 8 )^ Therefore, the similarities in the clinical presentations of these two diseases can lead to underdiagnosis or misdiagnosis. Besides, there may be a delay in diagnosis and treatment, worsening the clinical outcomes for both diseases. The diagnostic delay due to symptom similarity of COVID-19 and other infectious diseases has also been reported in other countries.^( 13 )^

Some studies have shown the onset of COVID-19 also brought an increase in the use of antibiotics, most of which are also employed for treatment of typhoid fever ( *e.g* ., azithromycin).^( 6 , 14 )^ This has further contributed to the increase in antimicrobial resistance, a significant threat to national health security.

In addition, the lack of facilities and laboratory expendables limits the capacity of typhoid fever testing.^( 5 , 15 )^As a result, antibiotics can be prescribed without laboratory confirmation, ultimately exaggerating antimicrobials resistance in the country. Limited access to COVID-19 testing in some regions of the country make the differentiation between COVID-19 and typhoid fever more challenging. These difficulties can compromise the treatment of both diseases, epidemiological surveillance efforts, and can also make the cases of COVID-19 and typhoid coinfection go unnoticed. Furthermore, diagnostic and treatment delays can lead to the deterioration of the clinical picture in both cases, which may require other complementary tests, challenging the resilience of the country’s health systems and increasing the burden in the health facilities and human resources.

Although it is not possible to evaluate how COVID-19 has exacerbated typhoid epidemiologically in Nigeria due to the lack of research, data and epidemiology surveillance programs, it is undeniable that COVID-19 has created a fertile environment for the spread of the disease throughout the country, especially in the poorest regions. Since there is still the possibility of a third wave of the COVID-19 pandemic around the world, measures must be taken to prevent the new wave of cases from affecting even more the country’s fragile health system.

To mitigate the situation and improve the diagnosis and management of typhoid fever, it is necessary to improve surveillance programs to address the existing lack of data in order to be able to understand the epidemiological situation of typhoid in the country, and to allow the development of an adequate response to both diseases, detect the areas at higher risk of the disease, promote prevention measures, and increase awareness through campaigns. The government and health bodies also need to improve laboratory facilities and deploy more typhoid fever testing facilities, while training healthcare workers in proper usage and handling of these facilities. This is necessary to improve Nigerian health systems ability to diagnose the disease, which can decrease the number of unreported cases.

Restriction on the sale of antibiotics as over-the-counter medications, awareness campaigns to educate the public about the risk of self-medication are also necessary. To prevent the spread of the disease, potable water should be made available for citizens through the Water Board Corporations in each state and efficient sewage management systems should be adopted particularly in urban areas with large populations. In addition, accelerating the rollout of typhoid vaccines could help protect the vulnerable groups and the population of the country in general.

The COVID-19 pandemic provides an opportunity to improve the prevention, diagnosis and management of typhoid fever in Nigeria. For instance, a multidisciplinary approach is necessary and can be done through combining health awareness campaigns aimed at the general public, continuous epidemiological surveillance, early detection of cases, training health care workers, increasing vaccination efforts, providing potable water, and promoting sanitation and hygiene programs. Only with this Nigeria will be able to lighten the burden of typhoid and COVID-19, and respond effectively against them, even in an eventual third wave of the COVID-19.
