# Supplementary material for: Typhoid fever and COVID-19 pandemic in Nigeria: a call for coordinated action
Source: Einstein (Sao Paulo). 2021 Dec 15;19:eCE6796. doi: 10.31744/einstein_journal/2021CE6796 (PMC8687648; doi:10.31744/einstein_journal/2021CE6796)
Supplement: Translation

# Translation: Febre tifoide e pandemia da COVID-19 na Nigéria: um apelo para uma ação coordenada

**Authors:** Esther Edet Bassey, Mohammad Mehedi Hasan, Ana Carla dos Santos Costa, Christos Tsagkaris, Abdullahi Tunde Aborode, Ayah Karra-Aly, Mohammad Yasir Essar, Shoaib Ahmad

**Affiliations:** 1 Nigeria Faculty of Basic Medical Sciences, University of Uyo, Uyo, NG, Nigeria.; 2 Bangladesh Faculty of Life Science, Mawlana Bhashani Science and Technology University, Tangail, BD, Bangladesh.; 3 Brasil Faculdade de Medicina, Universidade Federal da Bahia, Salvador, BA, Brasil.; 4 Greece School of Medicine, University of Crete, Heraklion, GR, Greece.; 5 Nigeria Healthy Africans Platform, Research and Development, Ibadan, NG, Nigeria.; 6 Canada Schulich School of Medicine and Dentistry, University of Western Ontario, London, CA, Canada.; 7 Afghanistan Medical Research Center, Kateb University, Kabul, AF, Afghanistan.; 8 Pakistan Punjab Medical College, Faisalabad, PK, Pakistan.

Prezado Editor,

A febre tifoide, também conhecida como febre entérica, é um problema significativo de saúde pública em países de baixa e média renda (PBMRs).^( 1 )^De acordo com a Organização Mundial da Saúde (OMS), aproximadamente 11 a 20 milhões de pessoas em todo o mundo adoecem por causa da febre tifoide, com mortalidade de 128 mil a 161 mil indivíduos por ano.^( 2 )^A doença, causada pela bactéria *Gram* -negativa *Salmonella typhi* , é transmitida por meio do consumo oral de uma porção patogênica da bactéria, na maioria das vezes em alimentos ou água infectados.^( 3 )^Também pode ser disseminada por meio de contato pessoal, devido a práticas não higiênicas, e pela contaminação do abastecimento de água.^( 4 )^

Devido às formas como a doença é transmitida, os PBMRs com problemas de saneamento de água, condições precárias de vida e práticas não higiênicas são mais suscetíveis a surtos de febre tifoide.^( 4 )^Na Nigéria, podem-se citar alguns fatores, como migração regional de trabalhadores, falta de sistema apropriado de tratamento de esgoto, abastecimento inadequado de água potável, alta taxa de migração rural para áreas urbanas e instalações de saúde insuficientes.^( 5 )^

Devido à ausência de um sistema de vigilância epidemiológica em todo o país e pela disponibilidade inadequada de dados e pela capacidade limitada de laboratórios, é difícil avaliar a prevalência da febre tifoide na Nigéria. Essa subnotificação também é causada por alguns fatores, como a administração de antibióticos antes do teste laboratorial de confirmação e o fato de que os pacientes nem sempre procuram tratamento médico nos hospitais, ou, ainda, utilizam os serviços laboratoriais, pois não são gratuitos. Entretanto, estudos anteriores documentaram prevalência entre 0,071%, em Oyo, e 47,1%, em Osun.^( 5 )^

Além de proporcionar acesso à água potável e melhorar as instalações sanitárias e de saúde, a vacinação é uma das melhores maneiras de combater a febre tifoide. Existem duas vacinas internacionais comercialmente disponíveis para a doença, que são seguras para uso em crianças – o grupo com maior risco de infecção por *S. typhi* . De fato, foi demonstrado que o uso de vacinas contra a febre tifoide em crianças com mais de 2 anos pode diminuir o risco de transmissão dessa doença.^( 6 )^Apesar das vantagens, as vacinas contra a febre tifoide não foram incorporadas ao *Expanded Program on Immunization* da Nigéria, representando um ônus extra no combate à doença.^( 5 )^

Como esperado, a pandemia da doença pelo coronavírus 2019 (COVID-19) teve um impacto negativo no controle de doenças infecciosas em todo o mundo, especialmente no esgotamento dos sistemas de saúde.^( 7 - 9 )^Isso aconteceu principalmente pelo aumento da demanda por recursos humanos, laboratórios e infraestrutura durante a pandemia da COVID-19, que desviou esforços antes destinados a outras doenças. A situação não foi diferente na Nigéria. Até 30 de abril de 2021, 164.993 casos tinham sido confirmados, com 2.063 mortes registradas no país.^( 10 )^Em uma tentativa de combater a doença, o governo nigeriano adotou estratégias aprovadas no mundo todo, como *lockdowns* , uso de máscaras, distanciamento social e lavagem de mãos. Entretanto, por mais eficazes que essas abordagens sejam, houve negligência na luta contra inúmeras doenças infecciosas preexistentes no país, e a pandemia aumentou a carga já existente no sistema de saúde do país.^( 11 )^

A situação é mais desafiadora, pois vários sintomas da febre tifoide são semelhantes à apresentação clínica da COVID-19.^( 8 , 12 )^Por exemplo, a febre tifoide geralmente tem sintomas que variam do início lento de febre contínua, calafrios, aumento do fígado e do baço (hepatoesplenomegalia) e dor abdominal, até anorexia, erupção cutânea, dor de cabeça, diarreia ou prisão de ventre, náusea, bradicardia relativa e baixo nível de consciência.^( 12 )^Em comparação, os sintomas da COVID-19 incluem febre, dor de cabeça, falta de ar, artralgia, dor de garganta, fadiga, dor no peito, hipogeusia, hiposmia, tosse, entre outros.^( 8 )^Portanto, as semelhanças nas apresentações clínicas dessas duas doenças podem levar a subdiagnósticos ou a diagnósticos errôneos. Além disso, pode haver atraso no diagnóstico e no tratamento, piorando os resultados clínicos de ambas as doenças. O atraso no diagnóstico devido à semelhança dos sintomas da COVID-19 e outras doenças infecciosas também foi relatado em outros países.^( 13 )^

Alguns estudos mostraram que o início da COVID-19 também trouxe aumento no uso de antibióticos – a maioria dos quais também é usada para o tratamento da febre tifoide (por exemplo, a azitromicina).^( 6 , 14 )^Isso contribuiu ainda mais para o aumento da resistência antimicrobiana, que é uma ameaça significativa à segurança sanitária nacional.

Além disso, a falta de instalações e de insumos laboratoriais limita a capacidade de testes para febre tifoide.^( 5 , 15 )^Consequentemente, os antibióticos podem ser prescritos sem confirmação laboratorial, resultando em aumento extraordinário da resistência aos antimicrobianos no país. O acesso limitado aos testes da COVID-19 em algumas regiões do país torna a diferenciação entre COVID-19 e febre tifoide ainda mais desafiadora. Essas dificuldades podem comprometer o tratamento de ambas as doenças e os esforços de vigilância epidemiológica e também podem fazer com que os casos de coinfecção por COVID-19 e febre tifoide passem despercebidos. Além disso, os atrasos no diagnóstico e no tratamento podem levar à deterioração do quadro clínico em ambos os casos, o que pode exigir outros exames complementares, desafiando a resiliência dos sistemas de saúde do país e aumentando a carga nos estabelecimentos de saúde e nos recursos humanos.

Embora não seja possível avaliar como a COVID-19 exacerbou, em termos epidemiológicos, a febre tifoide na Nigéria, por falta de pesquisas, dados e programas de vigilância epidemiológica, é inegável que a COVID-19 criou um ambiente fértil para a propagação da doença em todo o país, especialmente nas regiões mais pobres. Como ainda existe a possibilidade de uma terceira onda da pandemia da COVID-19 em todo o mundo, medidas devem ser tomadas para evitar que a nova onda de casos afete ainda mais o frágil sistema de saúde do país.

Para mitigar a situação e melhorar o diagnóstico e o tratamento da febre tifoide, é necessário melhorar os programas de vigilância, para resolver a falta de dados existentes, a fim de poder entender a situação epidemiológica da febre tifoide no país, permitir o desenvolvimento de uma resposta adequada a ambas as doenças, detectar as áreas de maior risco da doença, promover medidas de prevenção e aumentar a conscientização por meio de campanhas. O governo e os órgãos de saúde também precisam melhorar as instalações laboratoriais e implantar mais locais para testes de febre tifoide, ao mesmo tempo em que treinam os profissionais de saúde para uso e manuseio adequados dessas instalações. Isso é necessário para melhorar a capacidade dos sistemas de saúde nigerianos para diagnosticar a doença, o que pode diminuir o número de casos não notificados.

As restrições de venda de antibióticos sem retenção de prescrição e também as campanhas de conscientização para educar o público sobre o risco da automedicação são necessárias. Para evitar a disseminação da doença, água potável deve ser disponibilizada aos cidadãos por meio das empresas de abastecimento e saneamento em cada estado, e sistemas eficientes de tratamento de esgoto devem ser adotados, particularmente em áreas urbanas com grandes populações. Além disso, a aceleração do lançamento de vacinas contra a febre tifoide poderia ajudar a proteger os grupos vulneráveis e a população do país em geral.

A pandemia da COVID-19 oferece uma oportunidade para melhorar a prevenção, o diagnóstico e o tratamento da febre tifoide na Nigéria. Por exemplo, uma abordagem multidisciplinar é necessária e pode ser feita por meio da combinação entre campanhas de conscientização de saúde direcionadas ao público em geral, vigilância epidemiológica contínua, detecção precoce de casos, treinamento de profissionais de saúde, aumento das iniciativas de vacinação, fornecimento de água potável e promoção de programas de saneamento e higiene. Somente assim a Nigéria deve ser capaz de aliviar a carga da febre tifoide e da COVID-19 e agir de forma efetiva contra elas, mesmo com uma eventual terceira onda da COVID-19.
